# Experiences of adolescents and young adults with ADHD in Hong Kong: treatment services and clinical management

**DOI:** 10.1186/s12888-015-0478-x

**Published:** 2015-05-01

**Authors:** Kerry KW Cheung, Ian CK Wong, Patrick Ip, Phyllis KL Chan, Candy HY Lin, Lisa YL Wong, Esther W Chan

**Affiliations:** Centre for Safe Medication Practice and Research, Department of Pharmacology and Pharmacy, Li Ka Shing Faculty of Medicine, University of Hong Kong, Hong Kong, China; Department of Paediatrics and Adolescent Medicine, University of Hong Kong, Queen Mary Hospital, 102 Pok Fu Lam Road, Hong Kong, China; Department of Psychiatry, Child and Adolescent Psychiatric Team, Queen Mary Hospital, 102 Pok Fu Lam Road, Hong Kong, China

**Keywords:** ADHD, Adolescents, Adults, Qualitative, Experiences, Treatment, Impairment

## Abstract

**Background:**

Specialist services for the treatment of attention deficit hyperactivity disorder (ADHD) in adulthood in Hong Kong are yet to be developed. This study aims to explore the experiences of adolescents and young adults with ADHD in accessing treatment and services, coping with ADHD-related impairment, and their expectations of future treatment in Hong Kong.

**Method:**

Qualitative interviews were conducted with a semi-structured guide. Forty young adult patients aged between 16 and 23 were included in the study. The interview recordings were transcribed verbatim and anonymised. Data were analysed with a thematic approach based on key principles of Grounded Theory.

**Results:**

Four meta-themes were developed: Accessing ADHD diagnosis and treatment services; ADHD-related impairment; Experience of ADHD treatments; and Attitudes and expectations of future ADHD treatment. The role of parents and schools were highly significant in accessing services for patients diagnosed with ADHD in childhood. In general, ADHD affected every aspect of patients' lives including academic outcome, employment, family and social relationships. Medications were the principal treatment for ADHD amongst the interviewees and were reported to be generally effective. Half of the patients received non-pharmacological treatments in childhood but these effects were reported to be temporary. There was general consensus that the needs of patients with ADHD could not be met by the current service. In particular, there is a lack of specialist service for adults with ADHD, follow-up by different clinicians, and insufficient provision of non-pharmacological treatments.

**Conclusion:**

The findings suggest that further development of specialist ADHD services and non-pharmacological options for young adults are essential to meet their diverse needs with a holistic approach.

## Background

Attention deficit hyperactivity disorder (ADHD) is a common childhood-onset neurodevelopmental condition with a global prevalence between 5.9 and 7.1%, characterised by inattention, hyperactivity and impulsiveness [1]. The prevalence of ADHD in Hong Kong is estimated to be 6.1% in childhood and 3.9% in early adolescence [2]. ADHD often leads to behavioural problems at school, in family, social and work settings, and if left untreated, the effects tend to persist in adulthood [3,4]. The Diagnostic and Statistical Manual of Mental Disorders 5 (DSM-5), published in May 2013, addresses the needs of adults with ADHD [5]. This indicates an increasing worldwide focus on ADHD in recent decades, especially for those affected in adulthood.

ADHD can cause significant disruption to the lives of patients and their families. A previous study reported that delayed diagnosis, (after the age of 16), may cause more severe impairment, a sense of failure and missed potential in many areas of life [6]. Early diagnosis and intervention is important to offset the more severe effects in late adolescence/early adulthood. The brunt of the negative impact falls on academic performance and outcome while inappropriate social skills may undermine interpersonal relationships [6,7]. Adults with ADHD are more likely to experience emotional issues such as low self-esteem, lack of confidence, depression and anxiety [7,8]. Previous studies reveal that ADHD is associated with poor workplace performance, lower job status, and job stability [9-11].

In Hong Kong’s mental health services in particular, a rapid increase in the number of psychiatric outpatients since 1996 has been reported to adversely affect the quality of care [12,13]. Factors adding to the strain on the mental health system include a shortage of mental health staff and specialists, inadequate collaboration between current mental health institutions, and insufficient funding [14].

Patients under 18 years with ADHD in Hong Kong can be referred to public mental health services at paediatric clinics and child and adolescent psychiatric clinics under the Hospital Authority. Referrals can be made by various professionals such as school personnel, general practitioners, private psychiatrists, and psychologists from non-governmental organisations, child assessment centres and student health services from the Department of Health. The waiting time for the first appointment depends on patients’ conditions and emergency, ranging from several months to over a year. The major treatments for ADHD in Hong Kong are medication and psychotherapy. Medications include stimulants such as methylphenidate while non-stimulants include atomoxetine, imipramine and clonidine. Non-pharmacological treatments which predominantly focus on children with ADHD include psychoeducation, parent management training, simulated classroom behavioural training, impulse control and social skills training, executive function (EF) based training on homework; and self-management organised by hospitals, non-governmental organisations and primary schools.

In Hong Kong, schools and parents play a significant role in facilitating access to mental health services for children with ADHD. However, previous studies suggest that diagnosis and treatment of ADHD may be influenced by the parents’ decision to seek care and their awareness and knowledge about ADHD [15-17]. The key treatments for ADHD are medication and psychotherapy [18-20], which are focused on children, particularly the latter. In Hong Kong, ADHD diagnosis and treatment in adulthood is based primarily on the criteria specified in the Diagnostic and Statistical Manual of Mental Disorders (DSM) or International Classification of Diseases (ICD 10). Unlike countries such as the Netherlands, Australia and UK, where guidelines for ADHD in adulthood already exist [21-23], specialist services or a treatment pathway for adults with ADHD in Hong Kong is yet to be developed. It is necessary, therefore, to explore whether Hong Kong needs to develop a specialist treatment system to offer more comprehensive services to young adults with ADHD.

To our knowledge, no qualitative research has been carried out to explore the healthcare and treatment related experiences of adolescents and young adults with ADHD in Hong Kong and mainland China. This research is important as it opens up dialogue between service providers and users, enabling patients to express their needs and experiences, and identifies the gaps in current mental health services for ADHD. The study aimed to explore patients’ experiences: a) in accessing services; b) of pharmacological and non-pharmacological treatments; c) of ADHD-related impairments; and d) to ascertain their expectations of future services.

## Method

### Design

Qualitative interviews were used to explore patients’ experiences and opinions towards ADHD treatment in Hong Kong. According to a previous study, qualitative health research contributed to inform mental health policy and generated a better understanding of patients’ experiences and their active management of the disorder [24]. Such research is also timely since qualitative healthcare studies are scant. Due to the sensitive and potentially distressing nature of the questions, face-to-face interview was considered ideal for conducting the research. This also enabled the researcher to elicit appropriate information on unanticipated and related issues.

### Recruitment and participants

Ethics approval was obtained from the Institutional Review Board of the University of Hong Kong/Hospital Authority Hong Kong West Cluster (HKU/HA HKW IRB Number: UW13-197). Data collection was conducted between June 2013 and January 2014. Participants were eligible if they were diagnosed with ADHD in Hong Kong, were 16 years old or above, and had previous or current experience of pharmacological treatment for at least three months. Patients were excluded if they were unable to participate in a face-to-face interview. A total of 40 patients with ADHD were recruited and they were categorised into the following subgroups:

**Group 1:** Twenty patients aged between 16 and 17 years diagnosed with and receiving pharmacological treatment for ADHD in Hong Kong

**Group 2:** Twenty patients aged 18 years or above diagnosed with and receiving pharmacological treatment for ADHD in Hong Kong

Through this grouping method, this study was able explore patients’ expectation towards transition and future treatment in participants aged between 16 and 17 years. Recruitment of a separate group aged 18 years or above would enhance our understanding of the circumstances surrounding continued treatment in the child and adolescent psychiatry setting and the possible arrangement for future care. We adapted and translated interview questions from our earlier study conducted in the United Kingdom (UK) [6], designed to address the study hypotheses. The sample size was considered sufficient for qualitative research based on theme saturation in this study. Within each group, theoretical sampling, one of the three key steps of Grounded Theory, which refers to the continued process of sampling with regard to concepts built upon the findings analysed, was used.

Participants were recruited through two major methods. First, patients were recruited through the Department of Paediatrics and Adolescent Medicine, University of Hong Kong. From the list of potential participants retrieved by the department, patients were contacted by telephone and invited to attend a face-to-face interview. Second, patients were recruited through the Child and Adolescent Psychiatric Team at Queen Mary Hospital. Data on potential participants with follow-up appointments from November 2013 to January 2014 were retrieved from the Clinical Data Analysis and Reporting System of the Hospital (electronic patient record system) [25]. Those eligible were invited to participate in the study as they waited to be seen at clinic or were approached afterwards. Therefore, potential participants’ records of ADHD diagnosis and treatment were confirmed through the electronic patient record system.

### Procedure

Face-to-face interviews were carried out either in a consultation room at Queen Mary Hospital, interview room of the University of Hong Kong or other convenient locations for the subjects to attend. The semi-structured interview guide from an earlier study [6] was modified by a multi-disciplinary team, including two psychiatrists (PKLC & CHYL), two pharmacists (ICKW & EWYC) and a paediatrician (PI) to ensure the guide was relevant to the situation in Hong Kong. The interview questions included experiences of initial diagnosis and accessing services, ADHD-related impairments, ADHD treatments and expectations of future treatment. To ensure validity, the modified interview guide was piloted in six subjects to evaluate the relevance and suitability of the interview questions. Written informed consent was obtained before the interview. For those participants aged between 16 and 17, parental consent was also collected. Permission was obtained to digitally record the interviews, which lasted approximately 30 minutes to an hour and were conducted in Cantonese. Field notes were also made on participants’ thoughts, feelings and comments about the interview process.

Participants also completed a questionnaire on the demographics, current or most recent medication, symptoms and impairment severity. The Adult ADHD Self Report Scale (ASRS), a validated questionnaire was used to assess ADHD symptoms severity with 18 questions about adult ADHD symptoms and impairments [26]. In a separate impairment checklist from an ADHD clinical workbook [27], participants were asked to rate the extent of impairment (on a scale of 1–5, with higher scores indicating greater impairment), and how it impacts aspects of daily life such as family relationships, socialising, study, and work.

### Data analysis

The interview recordings were transcribed verbatim and anonymised. Data analysis was based on the key principles of Grounded Theory, which refers to an inductive methodology and possesses features of theoretical sampling, constant data comparison, memo writing, developing core categories and theoretical saturation [28]. Each interview transcript was analysed and key points were coded line-by-line, generating initial descriptive codes as data collection got underway. The additional codes from different transcripts contributed to interpretative analytical codes. Through the development of these codes, sub-themes and meta-themes were categorised and organised. It was an iterative process in that the study constantly revisited the transcripts as new themes emerged, and made comparisons between these themes [29]. Data collection and analysis were conducted simultaneously so any initial themes and unanticipated issues from existing data could be followed up in subsequent interviews [29]. Through the process of systematic analysis and comparison across the data set, several meta-themes were developed.

During the entire research process, field notes were made to record any thoughts and comments arising from each interview and during data analysis [30]. It was important for the researcher to be reflective as personal biases and pre-existing views might confound the research process and results [30]. Face and concept validation was undertaken in six pilot interviews to evaluate the relevance and suitability of interview questions and to review any problems and issues that arose during interview.

## Results

In the sample, 20 patients aged between 16 and 17 years (including 18 on continuous treatment and 2 who received medication treatment but had discontinued at the time of study) and 20 patients aged 18 years or above (including 16 on continuous treatment and 4 who received medication treatment but had discontinued at the time of study) were included through recruitment notice (n = 4), the Department of Paediatrics and Adolescent Medicine, University of Hong Kong (n = 5) and Child and Adolescent Psychiatric Team at Queen Mary Hospital (n = 31). There were a total of 27 males and 13 females. The mean age was 18 years (range 18–23) among those aged 18 or above (n = 20). As expected, over 90% of participants were Chinese (n = 37). Table 1 summarizes other participant characteristics.

**Table 1:** **Characteristics of participants**

	**Aged 17 or below (n = 20)**	**Aged 18 or above (n = 20)**
	**N (%)**	**N (%)**
**Gender**		
Male	13 (65)	14 (70)
Female	7 (35)	6 (30)
**Age**		
16	13 (65)	-
17	7 (35)	-
18	-	11 (55)
19	-	5 (25)
20-21	-	2 (10)
22-23	-	2 (10)
**Ethnicity**		
Chinese	18 (90)	19 (95)
Other Asian	0 (0)	0 (0)
Other mixed	1 (5)	0 (0)
Non-Asian	0 (0)	0 (0)
Other	1 (5)	1 (5)
**Work status**		
Student	20 (100)	15 (75)
Full-time job	0 (0)	4 (20)
Part-time job	0 (0)	1 (5)
Unemployed	0 (0)	0 (0)
**Living arrangements**		
Live alone	0 (0)	(0)
Live with parents	20 (100)	19 (95)
Live with spouse	0 (0)	0 (0)
Live with children	0 (0)	0 (0)
Other	0 (0)	1 (5)
**Education status**		
Junior high school (F.1-F.3)	5 (25)	3 (15)
High school (F.4-F.7)	15 (75)	11 (55)
Associate degree/high diploma	0 (0)	1 (5)
Bachelor degree	0 (0)	5 (25)

The age of ADHD diagnosis fell between 3 and 16 years (mean age at diagnosis = 9, SD = 3.10). Over 90% of participants were prescribed methylphenidate (n = 37), while other types of medication (n = 3) such as atomoxetine and clonidine were infrequently prescribed. Treatment duration since diagnosis ranged between 2 and 16 years (mean = 9, SD = 2.88). About half of the participants had received non-pharmacological treatment while the other half never had this experience. Duration of medication ranged between 3 months and 12 years with a mean duration of 6 years (SD = 3.49). Medical and treatment characteristics of participants are shown in Table 2.

**Table 2:** **Medical and treatment characteristics**

	**Aged 17 or below (n = 20)**	**Aged 18 or above (n = 20)**
	**N (%)**	**N (%)**
**ADHD diagnosis**		
Childhood	20 (100)	15 (75)
Late adolescence/adulthood	-	5 (25)
**Non-pharmacological treatment**		
No treatment	10 (48)	12 (50)
ADHD training/behavioural therapy	8 (38)	10 (42)
Psychological counselling	2 (10)	1 (4)
Both treatments	1 (5)	1 (4)
**Medication use**		
Using medication	17 (85)	16 (80)
Discontinued medication*	3 (15)	4 (20)
Medication type**		
Methylphenidate	19 (95)	18 (90)
Atomoxetine	1 (5)	1 (5)
Clonidine	0 (0)	1 (5)
**Duration since diagnosis**		
1-2 years	0 (0)	1 (5)
3-4 years	2 (10)	2 (10)
5+ years	11 (55)	9 (45)
10-14 years	7 (35)	7 (35)
15+ years	0 (0)	1 (5)
Duration of medication*** **(n = 37)**		
Less than 1 year	2 (11)	0 (0)
1-2 years	5 (26)	3 (17)
3-4 years	1 (5)	4 (22)
5-9 years	7 (37)	7 (39)
10-14 years	4 (21)	4 (22)

*Discontinued for a period of 6 months or longer.

**Current or most recent medication.

***Missing data for 3 participants.

In the total sample, the mean symptom severity score was 1.88 (SD = 0.58, range 0.94-2.94), and that of impairment severity was 1.50 (SD = 0.52, range 0.2-2.4) out of a total of 4. The mean score was generated based on the participants’ ratings (never = 0, rarely = 1, sometimes = 2, often = 3 and very often = 4) of their symptoms and impairment severity. The higher the score, the more severe the symptom or impairment. Overall, the rating of mean symptom and impairment severity was ‘mild’. Patients aged 18 or above (n = 20) had higher mean scores of symptom severity of 2.06 (SD = 0.62, range 0.94-2.94) and impairment severity of 1.50 (SD = 0.52, range 0.2-2.4) than those aged 17 or below (n = 20) with mean scores of symptom severity of 1.64 (SD = 0.40, range 1.06-2.72) and impairment severity of 1.41 (SD = 0.40, range 0.6-2.1).

Based on the Adult ADHD Self Report Scale [26], participants’ ratings (never, rarely, sometimes, often and very often) of their symptoms and impairment severity were coded on four levels of severity; none (scored between 0 and 1), mild (scored between 1.1 and 2), moderate (scored between 2.1 and 3), and severe (scored between 3.1 and 4 according to the mean symptom and impairment score. For symptom severity, 3% reported none, 65% reported mild and 33% reported moderate. For impairment severity, 23% reported none, 65% reported mild, and 13% reported moderate.

Data saturation was achieved and no new meta-themes emerged after the initial 20 interviews. Four meta-themes were developed: Accessing ADHD diagnosis and treatment services; ADHD-related impairments; Experiences of ADHD treatments; and Attitudes and expectations of future ADHD treatments. These themes and sub-themes are illustrated with quotes from the participants.

### Theme 1: Accessing ADHD diagnosis and treatment services

#### Experiences of seeking help for ADHD

Prior to accessing mental health services, the parents and teachers of many patients noticed they had difficulty concentrating or were fidgety, which distracted the class and affected their academic results (*“My dad discovered it at the time. I couldn’t concentrate during class and failed to hand in homework”* P4). Most of the participants had difficulty recalling how they first accessed public mental health services. Parents and schools played a significant role in getting referrals for patients who were too young to participate in the process (*“School referred me. The school social worker helped me”* P24; *“My parents brought me to child psychiatry and I had no idea why…perhaps they wanted me to have better concentration”* P8).

For some participants, accessing mental health services was not considered particularly challenging (*“It was a long time ago. I’ve totally forgotten”* P29). Some explained that referral was not complicated because a parent or school had arranged it (*“Not difficult because school teacher and social worker helped”* P11). However, for some, it took a long time before they received their first appointment in the public mental health service due to high number of patients (*“The referral process was troublesome because the public hospital needed you to wait and many people were in the queue”* P15).

As ADHD patients reached adulthood, patients’ care continued with child and adolescent psychiatry because the doctor decided that was more appropriate (*“I don’t want a new doctor. The doctor said my case is special so she will continue to follow my case”* P5). Those transferred to adult psychiatric service because they required continuous care (*“They said they only treat adolescents and I am an adult so I need to go to adult psychiatry to continue treatment”* P17)*.* Otherwise, the follow-up of their cases were terminated (*“They called me and said I am 18 years old and need to go to adult psychiatry and asked whether I want continuous care. I declined”* P8).

### Theme 2: ADHD-related impairments

#### Problems and challenges of ADHD

In general, many participants had difficulty dealing with academic matters (*“I can only do revision after taking the medicines”* P49). Inability to listen in class, interrupting lessons, failure to complete homework and revision were common, and adversely affected academic performance (*“I disturbed the teacher in the lesson…the teacher had to deal with me and couldn’t continue the lesson”* P23).

The impact of ADHD on social life also appeared to be considerable. Constant interrupting behaviours affected relationships with classmates and teachers (*“School teachers treated me badly…many classmates thought I was like that [interrupting and irritable] so they bullied me”* P29) while some encountered difficulty in forming friendships.

Some adult patients encountered difficulties with employment (*“If I have to do something for a period of time, slowly and repeatedly, I will start wandering, get off track”* P14). A few participants recalled that carelessness and poor attention hindered work performance (*“If someone asks me to work on… like an accounting document, I will not be able to manage because it inolves too many details…it limits my working ability”* P13).

Similarly, ADHD disrupted family relationships *(“I was hyperactive when I was a child, I loved to hit my younger brother”* P6). Other impairments included difficulties with time management (*“[I am a deadline fighter, for example I’ll remember the task only the last week before deadline”* P48)*.* Patients also expressed feelings of inferiority because of ADHD (*“Sometimes I feel sad…others are normal but I have this illness”* P30). Several participants were not diagnosed until late adolescence, and experienced the affects early on (*“If I received treatment earlier, at least I wouldn’t suffer from emotional problems that much. I could have had a happier childhood”* P10).

### Theme 3: Experiences of ADHD treatments

#### Pharmacological treatment

Overall, ADHD medication was effective and satisfactory. Medication increased attention span and reduced hyperactivity levels, which enabled the management of daily activities, particularly studying and working (*“The medication helps to keep me controlled. Helps me focus on my homework”* P18). For some, however, medication was ineffective (*“I did not notice a difference”* P50)*.* Indeed, growing up was found to reduce symptom severity and it was easier to be attentive and self-controlled (*“Better attention and organisation…because of brain development, there was less hyperactivity”* P34). On the other hand, patients also experienced common side effects of ADHD medication such as reduced appetite, poor sleep quality, lethargy and antisocial feelings (*“I completely don’t want to eat after medication, even if I love that food”* P44). Many patients were worried that medication would damage their health but generally accepted doctors’ clinical decisions (*“I worry that it will cause harm to body but doctor said it won’t”* P51).

### Experiences of non-pharmacological treatments

About half of participants reported taking part in ADHD group training offered at the hospital when they were primary school students. At the training, groups of children with ADHD played games and attended classes that discussed school curriculum, emotional control or social skills (*“Sometimes about emotional control or other things related to ADHD”* P36). Most, however, were not sure how effective treatment was (*“I was quite mature when I was a child. Others were playing games happily but I thought it was stupid…and I think the improvement was not notable. Why waste time on it?”* P25). A few said that non-pharmacological treatment together with medication would be more effective (*“Medicine improves physical problems and concentration, and behavioural therapy focus on psychological need”* P13).

Some patients had no previous non-pharmacological treatment, explaining they had no such need (*“When I was young, they [doctors] have talked to my family and me and I said I might not need it because I was not that serious”* P53). Some stated that doctors did not offer such treatments while others were not even aware non-pharmacological treatments were available (*“I don’t even know this [treatment] exists. Doctors didn’t tell me”* P51).

### Relationship between patient and doctor

In general, the relationship between patient and doctor was acceptable (*“She [doctor] understands your problems and knows how to solve it”* P34). A few patients did not have specific impressions because they saw different doctors and were asked similar routine questions *(“No relationship with doctor at all…and he has not followed the case regularly so he just asks those routine questions”* P49). In general, doctors were the major treatment decision-makers, particularly when they were children (*“Doctor makes the decision. I’m not qualified”* P40), although they took on this responsibility as they matured (*“The doctor will ask me the effect after medications and then I can make decision”* P31).

### Theme 4: Attitudes and expectation towards future treatment

#### Problems of previous or current treatments

Being seen by a different doctor at each consultation made follow-up intermittent and impeded build-up of rapport (*“The doctor was different at each consultation…I think it’s difficult to understand the patient’s need”* P52). Also, the prolonged waiting time for each consultation was frustrating (*“Waiting time should be shortened…I was waiting for an hour”* P42). As patients reached adolescence and adulthood, psychological treatments were insufficient. A few said they received psychological services only occasionally, such as counselling by a psychologist (*“It was not easy to access psychological service, if you wanted, it will be very expensive”* P12). Indeed, a few said that the quality of follow-up was poor as doctors spent five to ten minutes asking them routine questions (*“The doctor, sometimes her questions are so brief, even more so than my answers …like a routine”* P25).

### Suggestions for improvement

Suggestions were made for more investment in mental health services to alleviate pressure on the public health system (*“If some people are urgent…[public health care system] can’t solve their problems”* P16). More assistance or services were requested to meet the psychological needs of patients with ADHD. Some urged that more be done to raise public awareness and understanding of ADHD as they related their experiences of discrimination or being teased (*“The prejudice of general public in Hong Kong is very deep…it is associated with violence when they think of mental illness”* P17). There was hope that public education would address the misconceptions and increase understanding of ADHD (*“If there is more public awareness, less people will delay their treatment”* P49).

Appropriate guidelines and training on how to manage ADHD children should be available to teachers and parents. Some patients did not receive any support from teachers (*“I knew she [teacher] was busy but I just wanted some help. Was that so hard?”* P17), while parents tried to control their behaviour with punitive measures (*“Should teach parents how to deal with [ADHD] children, not only by scolding”* P32). Suggestions were made that doctors should explain ADHD better, particularly during childhood, to help patients understand their condition and work with other professionals to address their issues (*“Let the children know more about this disorder…if I know what is happening to myself, the attitude, even the effect of the treatment will be different”* P31).

### Expectations of future treatment

Most patients preferred to continue treatment from current institutions because of familiarity with services (*“The main purpose is getting the medicines”* P33). Patients were ambivalent regarding transition into adult services. They thought that child and adolescent psychiatry would be most appropriate because doctors were better acquainted with their cases but the transition was subject to doctor’s arrangement (*“The doctor here knows my condition all along. If I am transferred, they may not know what disorder I have”* P24). It showed that patients hoped to have more regular follow-up regarding their conditions. A few said that doctors in adult psychiatry might be more experienced to deal with problems encountered in adulthood. Indeed, most patients hoped that non-pharmacological options, such as counselling, or small groups to learn to control emotions, practise social skills and share experiences, would be available in adulthood (*“I think I need it regarding my emotions…small group seems better, more people and more fun”* P42).

## Discussion

The findings showed that patients diagnosed with ADHD in childhood as expected had very limited involvement in accessing related services. This was normally arranged by significant others, namely parents and schools who have close interaction with the children. Previous studies confirm that parents and teachers were influential in the decision-making of treatment management [31,32]. In our study, most of those diagnosed with ADHD in late adolescence had accessed private mental health services, as they perceived a strong and urgent need to deal with ADHD, but transferred to public services for continuous care due to the cost of private services.

In general, the impairment of most concern was related to academic issues since the onset of ADHD often occurred around school age [33]. The problems frequently reported by patients were inattention in class, and inability to cope with course work, which affected academic results, and is corroborated by a previous study on college adjustment in China [34]. A satisfactory social life was sometimes difficult, particularly when patients were young and hyperactivity was more common. Research indicates that children with ADHD tend to be more insensitive and self-centred in interpersonal relationships [35]. Young adult patients encountered difficulties in employment due to inability to focus on detailed tasks and procrastination [9]. Family discord, poor time management, and feeling distressed about having ADHD were also some of the issues raised.

Overall, patients were satisfied with previous or current treatments. Similar to a previous study in the UK, medications were the main treatment for ADHD [36]. Our patients reported that medication was effective, despite common side effects such as poor appetite and lethargy. About half of patients had received non-pharmacological treatments previously, mostly in childhood. Most patients thought that the effects were short-lived and did not help them cope with the challenges in adulthood. A previous study in the United States discussed the practical problems of non-pharmacological options where access to such treatment for a longer period of time was challenging [37]. As patients reached late adolescence, treatments that met their psychological needs were very limited, emphasising the lack of non-pharmacological options.

Another issue arising from treatment experiences was the relationship between patients and doctors, which directly affected the quality of care. Efforts by health professionals were often valued but patients were critical of frequent changes of the doctor in charge of their cases, which affected communication and rapport. Doubts were raised about the effectiveness of consultations that were conducted in a cursory manner in ten to fifteen minutes. Explanations about ADHD and managing the condition were deemed inadequate, particularly for children. More insight into their condition might change attitudes and improve drug compliance [38].

Due to the lack of specialist services for young adults with ADHD, treatment was continued in child and adolescent psychiatry as doctors viewed this to be the most appropriate option. Suggestions were made to some patients that they transfer to adult psychiatry for continuous care. Others were worried that doctors in the ADHD adult psychiatry service might not have an in-depth understanding of their problems due to a lack of experience. In view of the above findings, there is a significant gap in specialist services for ADHD in adulthood, which warrants further development.

The findings also illustrate current problems regarding insufficient public awareness and understanding of ADHD, and inadequate promotion of existing guidelines and training available to parents and teachers. The public perceives the classification ‘mental illness’ as a very violent and dangerous condition, resulting in stigmatism and discrimination [39]. In this respect, patients articulated the need for more education from government or other organisations to address these misconceptions. A previous study suggested that social rejection and stigmatism faced by ADHD patients and their parents could be reduced with community outreach and public health programmes [40]. Parents and teachers should be appropriately trained and supported to assist children with ADHD to cope with challenges. Previous studies also suggested that positive parenting not only resulted in fewer conduct problems but appropriate training helped to relieve the stress of caregivers in coping with ADHD [17,41]. Social studies have drawn up guidelines for schools to manage students with ADHD and facilitate social and academic achievement [42].

**Figure 1:**
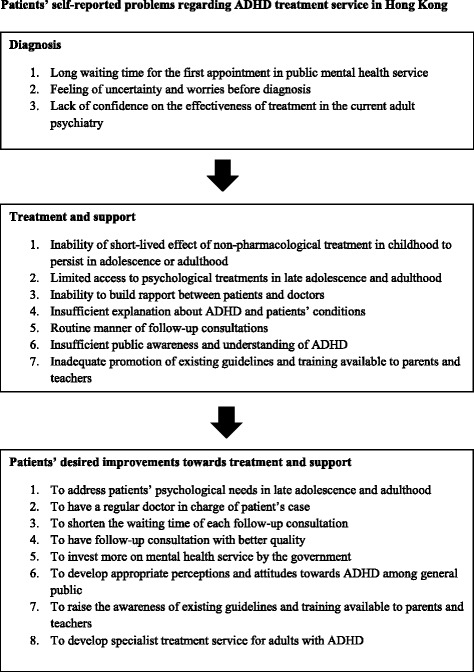
Patients’ self-reported problems regarding ADHD treatment service in Hong Kong.

As for future treatment in adulthood, most patients expected follow-up would continue at current mental health services where their cases were known. Although most were open-minded about transitioning to adult services, there was uncertainty and a lack of confidence on the effectiveness of treatment in the current adult service. These factors heighten the necessity for developing specialist services to address the gaps in continuity of care for young adults with ADHD. Figure 1 shows a summary of patients’ self-reported problems and desired improvements regarding ADHD treatment services in Hong Kong.

There were several limitations to the study. First, the sample size was small although it was deemed sufficient for qualitative study since generalising the findings was not the objective. With a small sample size of 40 participants, themes were not quantitatively derived. Moreover, the generalisability of the findings are limited and the participants to this study appear to be well managed based on the overall ‘mild’ self-reported symptoms and impairment severity. Indeed, participants’ experiences may be influenced by other comorbidities which were not accounted for in this study. In this study, we used the ASRS to rate their symptoms and impairments. Notably, the ASRS was developed as a screener for ADHD and the validity of the ASRS for capturing symptom burden rather than for indicating diagnosis is unknown. Second, the response rate was very low at the start of participant recruitment, which was conducted through telephone calls according to a patient list. With the recruitment strategy at the child psychiatric ADHD clinic, however, the response rate of 71% was much better. Patients were more inclined to participate in the research while waiting for their consultation. Additionally, many patients were unable to recall details of initial access to the services because they had little involvement in the process. Therefore, data collection in this section was insufficient.

This study suggests that future research should focus on the perspectives of parents, teachers, social workers, ADHD clinicians or other related parties in diagnosing and managing adults with ADHD current treatment modalities for ADHD and the transition of care of young adult patients in Hong Kong. Patients’ experiences of accessing private versus public services can be further investigated, as the organisation and quality of services are quite distinct. Further, the study included several patients who were diagnosed with ADHD in late adolescence and adulthood. Future study comparing the experience between patients diagnosed in childhood and adulthood in Hong Kong is useful when developing services.

Several implications for practice and policy can be drawn from the study through the four themes identified. First, the findings highlight the need to raise public awareness and understanding of ADHD to develop appropriate perceptions and attitudes among the general public. Increased public awareness of ADHD may help to minimise delayed diagnosis and reduce the impact of impairment. Since parents and teachers are in close daily contact with children with ADHD, appropriate guidelines, training and support are essential to facilitate communication. Therefore, awareness and promotion of these programmes are equally important. Additionally, this study underscores the need and increasing interest for more non-pharmacological treatment. Medication as the sole treatment for ADHD is prevalent as patients reach adulthood yet it is deficient in meeting their psychological needs. Consequently, there is a pressing need to develop specialist services to offer better and more specific support to young adults with ADHD. Importantly, the four identified themes suggest that individuals may be vulnerable to suboptimal care and these themes are important aspects to understand in the process of health system improvement.

## Conclusions

This study explores adolescents and young adults’ experiences of ADHD impairment, access to services, treatment, and their expectations of future treatment. Further improvements are required to meet the needs of adult patients such as more non-pharmacological treatment, and increasing public awareness and understanding of ADHD. Appropriate training for parents and teachers would be strategic in helping children with ADHD. As previous studies have shown, ADHD remains challenging in late adolescence and adulthood. These support the findings that specialist services for ADHD in adulthood are necessary as current adult psychiatry services may not meet patients’ needs. Further research is required to further understand the perspectives of involved parties such as parents, teachers, social care, and health care professionals to assess the unmet needs of those affected with ADHD, and to develop a multidisciplinary approach to their care.
